# Structural Basis of Artemisinin Binding Sites in Serum Albumin with the Combined Use of NMR and Docking Calculations

**DOI:** 10.3390/molecules27185912

**Published:** 2022-09-12

**Authors:** Alexandra Primikyri, Georgios Papamokos, Themistoklis Venianakis, Marianna Sakka, Vassiliki G. Kontogianni, Ioannis P. Gerothanassis

**Affiliations:** Section of Organic Chemistry and Biochemistry, Department of Chemistry, University of Ioannina, GR-45110 Ioannina, Greece

**Keywords:** artemisinin, serum albumin, STD NMR, STD-TOCSY NMR, TR-NOESY NMR, INPHARMA NMR, docking calculations

## Abstract

Artemisinin is known to bind to the main plasma protein carrier serum albumin (SA); however, there are no atomic level structural data regarding its binding mode with serum albumin. Herein, we employed a combined strategy of saturation transfer difference (STD), transfer nuclear Overhauser effect spectroscopy (TR-NOESY), STD–total correlation spectroscopy (STD-TOCSY), and Interligand Noes for PHArmacophore Mapping (INPHARMA) NMR methods and molecular docking calculations to investigate the structural basis of the interaction of artemisinin with human and bovine serum albumin (HSA/BSA). A significant number of inter-ligand NOEs between artemisinin and the drugs warfarin and ibuprofen as well as docking calculations were interpreted in terms of competitive binding modes of artemisinin in the warfarin (FA7) and ibuprofen (FA4) binding sites. STD NMR experiments demonstrate that artemisinin is the main analyte for the interaction of the *A. annua* extract with BSA. The combined strategy of NMR and docking calculations of the present work could be of general interest in the identification of the molecular basis of the interactions of natural products with their receptors even within a complex crude extract.

## 1. Introduction

Artemisinin and its semisynthetic derivatives are a group of medicines used to treat malaria due to *Plasmodium falciparum,* which have drawn considerable scientific interest in the last decades [1]. Tu’s discovery of artemisinin and dihydroartemisinin in 1969 was a significant breakthrough in 20th-century tropical medicine, saving millions of lives (Nobel Prize in Medicine, 2015). Since then, several studies have revealed information about the therapeutic value of artemisinin and its derivatives not only against malaria but also for other diseases, including cancers, inflammatory diseases, and autoimmune and neurodegenerative disorders [2,3,4]. 

Recently, the in vitro efficacy of artemisinin and its derivatives against SARS-CoV-2 strains was shown in human cell lines in an effort facing the global outbreak of COVID-19. Additionally, the pharmacokinetics and their plasma concentration−time profiles were studied to determine the optimum concentration that could be intravenously administered [5,6]. These studies provide potent leading candidates for anti-SARS-CoV-2 drug research and development. Artemisinin is known to bind to the main plasma protein carrier serum albumin (SA), which is involved in the absorption, distribution, metabolism, and excretion of drugs, with a binding constant of 2.38 × 10^4^ M^−1^ [7]. 

Human serum albumin (HSA) is a single-chain, non-glycosylated protein consisting of three homologous *α*-helical domains (I, II, and III) [8]. X-ray single crystal structure determination demonstrated that there are two binding sites, Sudlow’s I site (Drug site I) and Sudlow’s II site (Drug site II), which are located in the IIA and IIIA subdomain, respectively [9]. Serum albumin plays a significant role in the pharmacokinetics and pharmacodynamics of many drugs since most of them circulate in the plasma and reach the target tissues through binding to SA.

Thus far, there are only a limited number of studies investigating the interaction profile of artemisinin with human (HSA) and bovine (BSA) serum albumin. UV-Vis absorption and fluorescence spectral studies suggested the interaction of artemisinin with HSA [7,10]. Furthermore, synchronous fluorescence, three-dimensional fluorescence, and CD spectroscopy showed an alteration of the secondary structure of HSA in the presence of artemisinin [10]. 

In addition, docking calculations revealed that artemisinin can bind to Drug Site I of HSA with the formation of a hydrogen bond with Arg218 [7,10]. Similar studies with bovine serum albumin, which has a 76% amino acid homology and an overlapping folding with HSA, demonstrated a static quenching mechanism of binding and revealed one binding site located within site I, subdomain IIA [11,12]. Fluorescence competition experiments showed that artemisinin could displace the drugs bilirubin and ketoprofen from Drug site I but not ibuprofen and chlorphenamine maleate from Drug Site II, thereby, suggesting that artemisinin can only bind to subdomain IIA [12]. 

The chemical structure of artemisinin is characterized by the presence of an endoperoxide bridge, which is essential for its biological activity, and a δ-lactone ring [13]. Even though artemisinin has proven to be a valuable candidate within the drug development arsenal of scientists, there are no atomic level structural data of the interaction profile of the drug with its carrier human (HSA) and bovine (BSA), serum albumin. For this reason, herein, we employed a combined strategy of saturation transfer difference (STD), transfer nuclear Overhauser effect spectroscopy (TR-NOESY), STD–total correlation spectroscopy (STD-TOCSY) and Interligand Noes for PHArmacophore Mapping (INPHARMA) NMR methodologies, and molecular docking calculations to investigate the molecular basis of the interaction of artemisinin with BSA/HSA.

## 2. Results and Discussion

### 2.1. Epitope Mapping of Bound Artemisinin with the Combined Use of STD, STD-TOCSY, and Tr-NOESY NMR

The binding mode of artemisinin with BSA/HSA was initially investigated by employing STD NMR spectroscopy, which allows the identification of ligand moieties that interact with the binding site of a protein. The method is based on the fact that only the protons that interact and receive saturation transfer from the receptor, via spin diffusion through the nuclear Overhauser effect, will appear in the STD spectrum. Furthermore, those protons that are in close vicinity to the protein will demonstrate more intense STD signals due to a more efficient magnetization transfer [14,15]. 

**Figure 1:**
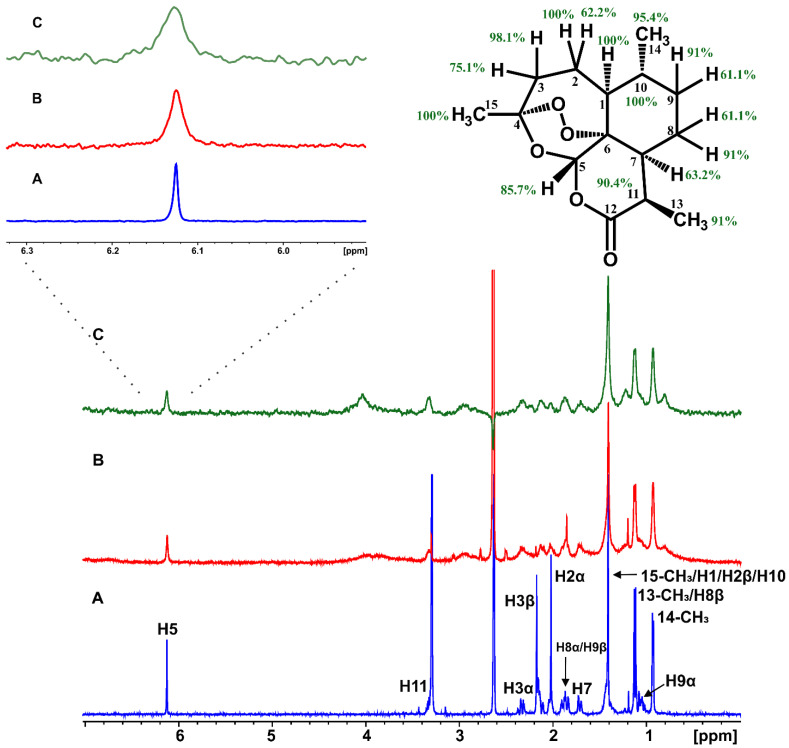
(**A**) ^1^H NMR of 2 mM artemisinin in PBS, pD 7.4, D_2_O with 10% DMSO-d_6_. (**B**) ^1^H NMR of 2 mM artemisinin with 20 μM BSA in PBS, pD 7.4, D_2_O with 10% DMSO-d_6_. (**C**) STD NMR of 2 mM artemisinin with 20 μM BSA. Τ = 310 K, number of scans = 320, experimental time = 3 h 30 min.

Artemisinin was added in excess in a BSA buffer solution with a ligand-to-protein ratio of 100:1 [16]. The STD NMR spectrum (Figure 1C) revealed that all the protons of artemisinin are involved in this interaction. Further evidence of this binding was also obtained from the significant line broadening and the increase in the transverse relaxation rate as a result of BSA addition (Figure 1A,B). Similar results were obtained with the interaction of artemisinin with HSA (Figure S1, Supplementary Materials).

STD NMR intensities allow the identification of artemisinin contacts with BSA in the complex. The epitope mapping of bound artemisinin was performed through the determination of the STD amplification factor (*A*_STD_) for each one of the interacting protons. Providing that all protons have similar relaxation rates, the differences in the relative STD response (*A*_STD_) for each proton reflect its relative proximity to the receptor-binding site. 

Having confirmed that the T_1_ longitudinal relaxation time of the slowest relaxing nuclei was 1.01 s, an STD build-up stack was obtained by varying the saturation times (0.25, 0.5, 1, and 2 s). The optimum saturation time of 2 s was selected to give the stronger STD signals. Group epitope mapping calculations were made by comparing the individual proton integrals and the relative *A*_STD_ with the highest intensity was set to 100%. All other STD signals were calculated and normalized with respect to this signal.

The efficient binding of artemisinin is illustrated in Figure 1 since all the protons show *A*_STD_ values above 60%. Although discrimination between the overlapping H1, 15-CH_3_, H10, and H2β protons cannot be achieved, proton H3a, which belongs to the 1,2,4-trioxane ring and is responsible for artemisinin’s mechanism of action, shows a major involvement in the interaction with an *A*_STD_ value of 98.1%. The 14-CH_3_ methyl group follows with an *A*_STD_ value of 95.4%, and proton H11 presents a value of 90.4%, thus, demonstrating strong binding to BSA. 

**Figure 2:**
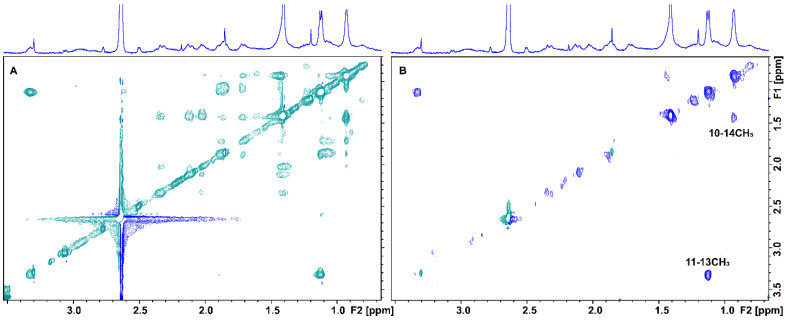
(**A**) 2D TOCSY NMR of 2 mM artemisinin and 20 μM BSA in PBS, pD 7.4, D_2_O with 10% DMSO-d_6_. (**B**) 2D STD-TOCSY NMR of 2 mM artemisinin with 20 μM BSA in PBS, pD 7.4, D_2_O with 10% DMSO-d_6_. Mixing time = 60 ms, T = 310 K, number of scans = 40, and experimental time = 4 h 20 min.

Proton H5 between the two oxygen groups, with the most deshielded resonance in the ppm scale, shows an *A*_STD_ value of 85.7% also indicating strong binding. Protons H7, H8α/H9β, H2α, and H3β show the lowest, but significant, *A*_STD_ values of 63.2, 61.1, 62.2, and 75.1%, respectively. To further resolve the overlapping signals and to investigate which protons were in closer contact with the protein surface, a two-dimensional STD total correlation spectroscopy (2D-TOCSY) spectrum of artemisinin bound to BSA was acquired. The more intense signals in the STD-TOCSY spectrum correspond to the more saturated protons and, therefore, are the closest to the receptor’s binding surface [17,18]. Figure 2A illustrates a standard TOCSY NMR spectrum compared to the respective STD-TOCSY spectrum (Figure 2B). Cross-peaks between protons H11/13-CH_3_ and H10/14-CH_3_ demonstrate strong STD-TOCSY NMR signals suggesting a closest approach to the binding sites and further validating the STD NMR results with *A*_STD_ values above 90%. Additionally, it is clarified that high *A*_STD_ values for the overlapping STD signals of 13-CH_3_/H9α/H8β and H1/15-CH_3_/H10/H2β protons can contribute to the STD effects of 13-CH_3_ and H10 protons and only to a lesser extent to the rest of the protons. It is of interest that the rest of the signals disappeared in the STD-TOCSY spectrum due to their lower degree of saturation.

**Figure 3:**
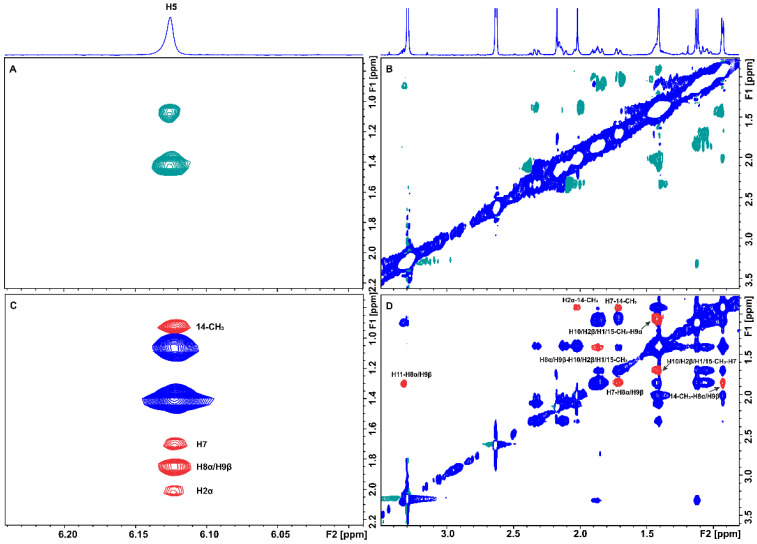
(**A**,**B**) Selective regions of 2D NOESY NMR spectrum of 2 mM artemisinin in PBS, pD 7.4, D_2_O with 10% DMSO-d_6_. (**C**,**D**) Selective regions of 2D Tr-NOESY NMR spectrum of 2 mM artemisinin with 20 μM of BSA in PBS, pD 7.4, D_2_O with 10% DMSO-d_6_. New Tr-NOE cross-peaks are denoted with red color. Mixing time = 300 ms, T = 310 K, number of scans = 112, and experimental time = 17 h 19 min.

Tr-NOESY experiments were also performed in order to investigate the bound conformation of artemisinin. A small MW ligand possesses a short correlation time (*τ_c_*) and a longer relaxation time in the free form, while in the bound state, it acquires the behavior of the large MW receptor and, thus, a longer correlation time (*τ_c_*). This results in strong negative tr-NOEs [19] that can be observed at the position of the free ligand, thus, providing important NOE distance information of the ligand in the bound state [15]. The 2D NOESY spectrum of artemisinin in PBS buffer solution is presented in Figure 3A,B. Several connectivities with opposite phase to that of the diagonal are observed due to protons with distances ≤ 5 Å. 

In the 2D Tr-NOESY spectrum of artemisinin bound to BSA, a significantly larger number of connectivities are observed with the same phase as that of the diagonal (Figure 3C,D). Strong tr-NOEs between protons, such as H11 and H8α/H9β, H7 and H8α/H9β, and 14-CH_3_ and H8α/H9β are observed, which are absent in the NOESY spectrum of the free ligand. Interestingly, new Tr-NOE peaks between proton H5 and protons 14-CH_3_, H7, H8α/H9β, H2α, H3α, and H11 are also observed, presumably due to an efficient spin-diffusion process in the bound state and because artemisinin adopts a more closed conformation within the binding cavity of BSA/HSA.

### 2.2. STD and INPHARMA NMR Competition Experiments of Artemisinin with Warfarin and Ibuprofen

**Figure 4:**
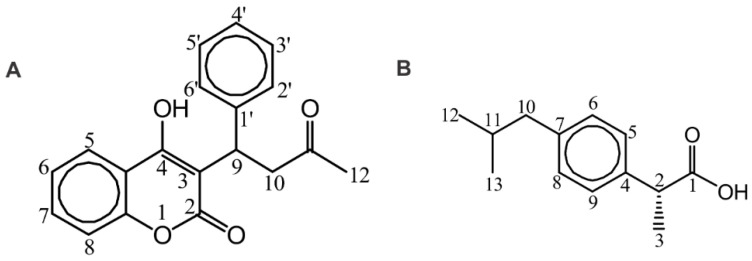
Chemical structures of warfarin (**A**) and ibuprofen (**B**) with the numbering of atoms.

Competition experiments were performed with warfarin and ibuprofen (Figure 4), which are two common BSA/HSA drugs. Crystallographic data reveal that warfarin binds to drug site 1 (in subdomain IIA) and that ibuprofen binds both to the center of the binding pocket of subdomain IIIA within drug site 2 and to a secondary site located at the interface between subdomains IIA and IIB at the base of the protein between drug site I (domain IIA) and drug site II (domain IIIA) [9,20,21,22,23]. Previous extensive studies of the complexation of warfarin with HSA showed a wide range of formation constants (10^4^–10^5^ M^−1^) depending on various experimental conditions [24,25,26,27,28,29].

**Figure 5:**
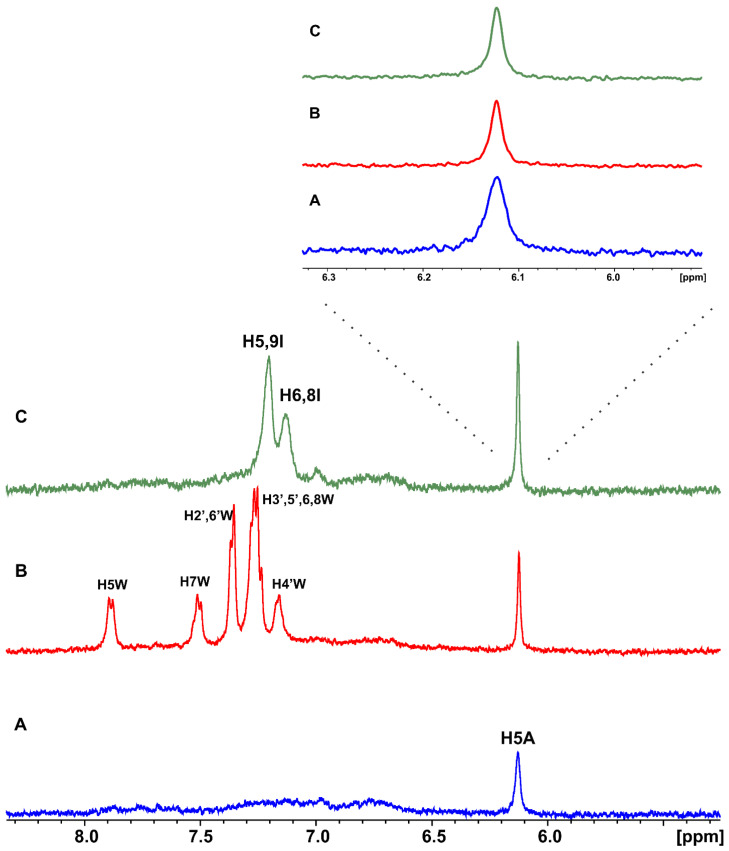
(**A**) Selected regions of ^1^H NMR of 2 mM artemisinin with 20 μM BSA in PBS, pD 7.4, D_2_O with 10% DMSO-d_6_, (**B**) after the addition of 2 mM warfarin and (**C**) after the addition of 1 mM ibuprofen.

Figure 5 and Figure S2 (Supplementary Materials) illustrate sharper (for example, H5) 1D NMR resonances upon the addition of warfarin (Figure 5B and Figure S2B (Supplementary Materials)) and ibuprofen (Figure 5C and Figure S2C (Supplementary Materials)) suggesting that they compete and replace artemisinin from common binding sites due to their higher binding affinity. As already mentioned, the STD NMR spectrum of artemisinin with BSA/HSA demonstrates that all protons of the ligand interact with the proteins (Figure 1 and Figure S1 (Supplementary Materials)). 

**Figure 6:**
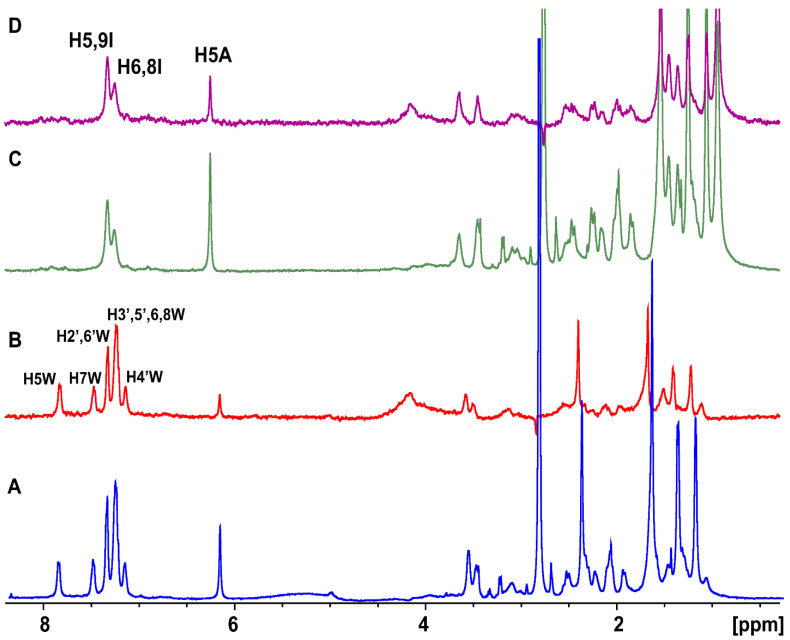
(**A**) ^1^H NMR of 2 mM artemisinin and 1.8 mM of warfarin with 20 μM of BSA in PBS buffer solution in D_2_O, pD 7.4 with 10% DMSO-d_6_. (**B**) STD NMR of sample A. (**C**) ^1^H NMR of 2 mM artemisinin, 1 mM of ibuprofen, and 20 μM of BSA in PBS buffer solution, pD 7.4 in D_2_O with 10% DMSO-d_6_. (**D**) STD NMR of sample C. Τ = 310 K, number of scans = 320, and experimental time = 3 h 30 min.

Interestingly, after the addition of an equimolar concentration of warfarin in the complex of artemisinin with BSA, the resulting STD signal integrals (warfarin/artemisinin 3/1) clearly demonstrate a stronger STD effect and, thus, a higher affinity of warfarin relative to that of artemisinin (Figure 6A,B). Similar results were obtained in the case of the addition of warfarin in the complex of artemisinin with HSA (warfarin/artemisinin 2:1), which resulted in a ratio of 6/1 in the respective STD NMR integrals (Figure S3A,B, Supplementary Materials). 

The same conclusion can be drawn for ibuprofen, which was added in a molar ratio of ibuprofen/artemisinin 1:2 in the complex of artemisinin with BSA (Figure 6C) and a ratio of 1/1 in the complex of artemisinin with HSA (Figure S3C, Supplementary Materials). Interestingly, the STD effects of ibuprofen are three to five times stronger compared to that of artemisinin (Figure 6D and Figure S3D (Supplementary Materials)). This suggests a significantly higher binding affinity of ibuprofen for BSA/HSA relative to that of artemisinin. 

The above linewidth and STD NMR results provide evidence that the interaction of artemisinin is competitive with warfarin and ibuprofen; however, the allosteric mechanism cannot be excluded. INPHARMA methodology can be used to distinguish between competitive and allosteric binding modes of a ligand to a macromolecular target if the binding mode of a second competitive ligand is known. The principle of the INPHARMA approach is based on the NOE magnetization transfer between two ligands that bind competitively to a common macromolecular receptor [30,31,32,33,34] provided that the inter-ligand distances are ≤5 Å. 

INPHARMA methodology was used in order to reveal the binding mode of artemisinin through competition with the two drugs, warfarin and ibuprofen, based on the observation of inter-ligand NOEs between artemisinin and warfarin/ibuprofen. 2D Tr-NOESY experiments were recorded at different mixing times (100, 200, and 300 ms) for the mixtures of artemisinin with BSA/HSA after the addition of warfarin. Inter-ligand NOE cross-peaks were observed between all the aromatic protons of warfarin and several of the protons of artemisinin (Table S1, Supplementary Materials). 

**Figure 7:**
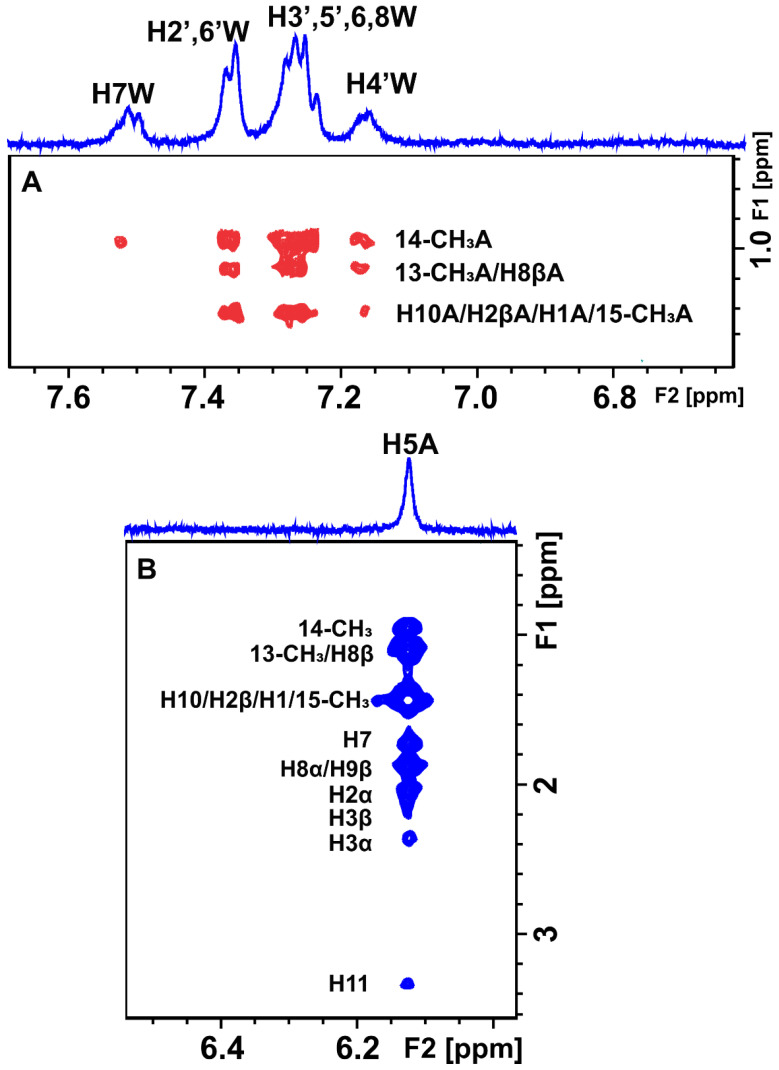
Selective region of the 2D Tr-NOESY NMR spectrum (d8 = 300 ms) of 2 mM artemisinin and 20 μM BSA after the addition of 2 mM warfarin in PBS buffer solution in D_2_O, pD 7.4 with 10% DMSO-d_6_. (**A**) Red cross-peaks correspond to inter-NOEs between warfarin and artemisinin and (**B**) blue cross-peaks correspond to intra-NOEs of artemisinin. NMR parameters are the same as in Figure 3.

In the case of HSA, all the aromatic protons demonstrated inter-NOEs with the H1, 15-CH_3_, H10, and H2β protons of artemisinin, which overlap. Whereas, in the case of BSA, the same aromatic protons of warfarin except for H-5 and H-7 demonstrated inter-NOEs with the 13-CH_3_ and H8β protons, which overlap in the ^1^H NMR spectrum and with H1, 15-CH_3_, H10 and H2β protons of artemisinin, which also overlap (Figure 7 and Figure S4, Supplementary Materials). Interestingly, the strong STD effects of these three peaks of artemisinin further confirm the conclusion that these protons are mainly responsible for this interaction.

**Figure 8:**
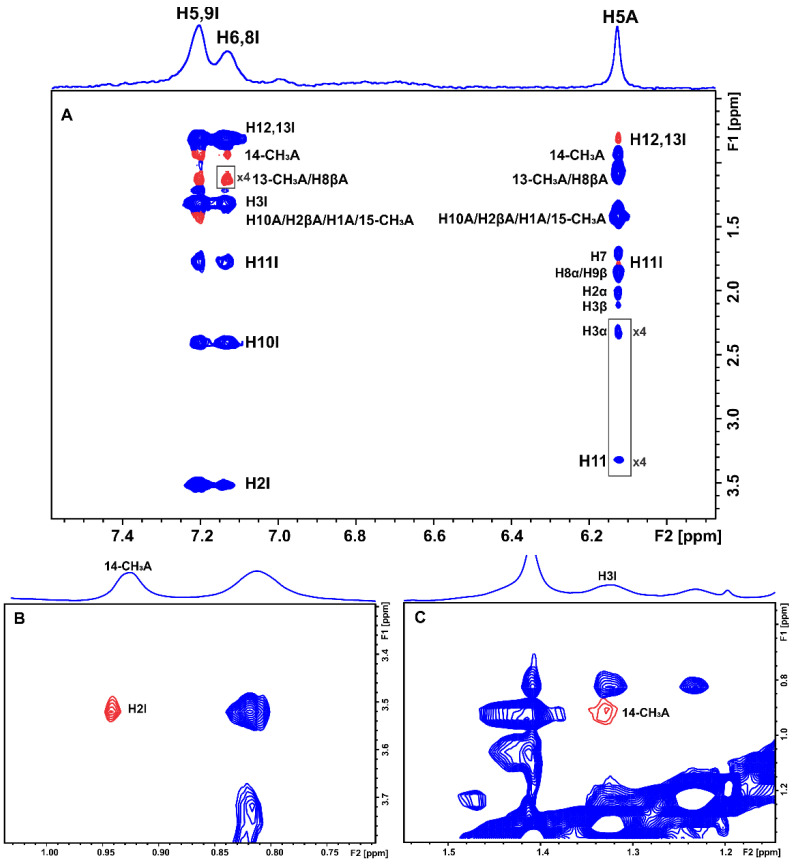
Selective regions of 2D Tr-NOESY NMR spectrum (d8 = 300 ms) of 2 mM artemisinin and 20 μM BSA after the addition of 1.2 mM ibuprofen in PBS buffer solution in D_2_O, pD 7.4 with 10% DMSO-d_6_. Blue cross-peaks correspond to intra-NOEs and red cross-peaks to inter-NOEs. For the notation of inter-NOEs in (**A**–**C**), see text. The NMR parameters are the same as in Figure 3.

INPHARMA methodology was also applied to the mixtures of BSA/HSA with artemisinin and ibuprofen. 2D NOESY experiments were recorded at different mixing times (100, 200, and 300 ms). Several inter-ligand NOEs were clearly observed between the two molecules corroborating the NOE transfer between the two ligands at the common BSA binding sites, drug site II (subdomain IIA), and drug site I in the interface between subdomains IIA and IIB. Inter-NOEs include cross-peaks between the aromatic protons of ibuprofen and the protons 14-CH_3_, 13-CH_3_, and H8β, which overlap, and protons H1, 15-CH_3_, H10, and H2β, which also appear at the same resonance (Figure 8A and Table S1 (Supplementary Materials)). 

In particular, the H5,9 aromatic protons of ibuprofen show a more intense inter-NOE with the 14-CH_3_ protons of artemisinin in comparison with the H6,8 aromatic protons. Proton H2 and the H3 of ibuprofen also demonstrate inter-NOEs with protons 14-CH_3_ of artemisinin (Figure 8B,C). On the other hand, the most characteristic resonance of artemisinin, proton H5, demonstrates an inter-NOE cross-peak with the protons H12 and H13 of ibuprofen at 0.82 ppm and a less intense inter-NOE with H11 at 1.77 ppm, which is absent in the respective spectrum with HSA (Figure S5, Supplementary Materials). 

The significant number of inter-ligand NOE connectivities provide unequivocal experimental evidence of a competitive, rather than allosteric, binding mode of artemisinin in the warfarin (FA7) [35] and ibuprofen (FA4) binding sites. These inter-ligand NOEs connectivities greatly facilitated our docking calculations (see Section 2.3). 

### 2.3. Docking Calculations

The strategy for the investigation of the atomic level interaction between artemisinin, warfarin, and ibuprofen with human serum albumin (HSA) was similar to that employed in our previous work on the interaction of unsaturated fatty acids with HSA/BSA [33]. The HSA crystal structure free of ligands was downloaded from PDB (entry code is 1BM0 [36]). The docking conformations were selected based on the highest binding affinity and in accordance with experimental STD, 2D NOESY, and, mainly, INPHARMA NMR data.

#### 2.3.1. Warfarin Binding Site FA7

The X-ray single crystal structure of warfarin with HSA shows that the binding site has two sub-chambers that accommodate the coumarin and the benzyl moieties of the drug [20]. The coumarin moiety binds to the main chamber of HSA, and the furthest one from the entrance of the binding site forms mainly hydrophobic interactions. The benzyl moiety fits into a sub-pocket also primarily through hydrophobic interactions. On the other hand, the acetonyl group is closer to the entrance of the binding pocket, and the oxygen atom contributes to a hydrogen bond interaction with R222.

For the warfarin binding site FA7, site-specific docking was performed with increased exhaustiveness for the following protein-ligand pairs: 1BM0–artemisinin and 1BM0–warfarin. The binding site FA7 (subdomain IIA) is characterized by the amino acids K199, R218, and R222 (anchor site I) and H242 and R257 (anchor site II) [33]. The affinity of warfarin at FA7 of HSA ranges between −8.7 and −7.6 kcal/mol for the nine most probable poses. In comparison, the affinity of artemisinin for the same binding site of HSA and the same search space ranges between −6.9 and −6.4 kcal/mol for the nine most probable poses. 

**Figure 9:**
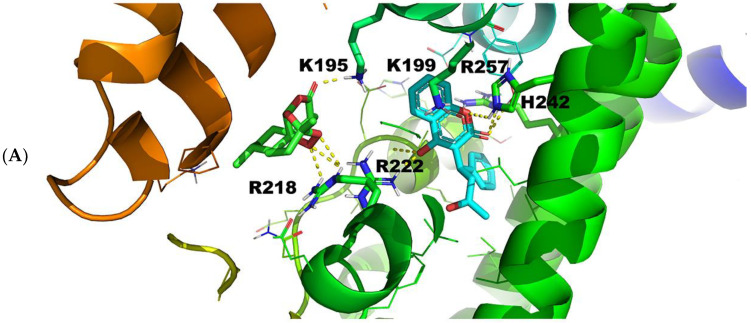

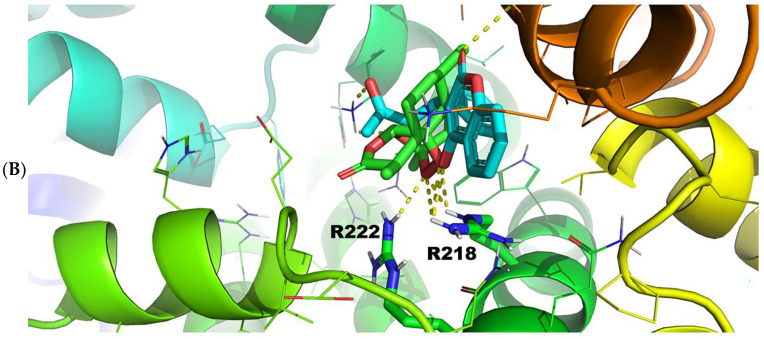
(**A**) Pose number 2 of warfarin and artemisinin for binding site FA7 of HSA (1BM0.pdb). (**B**) Superimposed pose number 2 of (**A**).

The docking results, therefore, are in excellent agreement with the significant reduction in the NMR linewidths (Figure 5A,B and Figure S2A,B (Supplementary Material)) and STD integrals (Figure 6A,B) of artemisinin resonances upon the addition of warfarin. The visualization of the results helps us to understand where this difference stems from: warfarin generates more interactions in FA7, as shown in Figure 9, due to the ability of this binding site to form two anchor sites of polar amino acids. 

Figure 9B (pose number 9) shows that, although both ligands interact mainly with amino acids R218 and R222, the interaction of warfarin is stronger by 1 kcal. This difference can be attributed to the ionic character of the interaction between the negatively charged warfarin and positively charged amino acids. Warfarin, therefore, shows more flexibility and can interact with a greater variety of amino acids simultaneously and at different spatial characteristics.

The results produced by the 2BXD structure showed that warfarin can replace artemisinin: the highest affinity for both ligands was found to be −8.3 kcal/mol. For artemisinin, however, only six poses were generated. The last four of the six had binding affinities lower than 6.7 kcal/mol. For warfarin, nine poses were recorded: four with binding affinities higher than 8.1 kcal/mol, while the rest were found to be higher than 6.7 kcal/mol. Thus, both crystal structures indicated that warfarin is the stronger competitor. These results are in excellent agreement with our STD NMR competition experiments (Figure 5, Figure 6, Figures S2 and S3). Furthermore, fluorescence experiments showed a binding constant of artemisinin to HSA of 2.38 × 10^4^ M^−1^ [7], which can be compared with a recent isothermal titration calorimetry value of 3.57 × 10^5^ M^−1^ of warfarin bound to HSA [33]. 

It would be of interest to compare selected poses of the docking calculations with experimental inter-ligand NOEs of Table S1. 14-CH_3_, 13-CH_3_, and 15-CH_3_ resonances show strong and strong-to-medium inter-NOEs, especially with the benzoyl moiety of warfarin. This is in excellent agreement with the superposition of pose number 2 of artemisinin and warfarin (Figure 9B), which shows that the respective functional groups have distances <0.5 Å. It is of interest that no inter-NOEs were observed between H-5 of artemisinin and the protons of warfarin. This is in excellent agreement with the docking results of Figure 9B, which show distances >5 Å and, thus, are beyond the limits of the NOE experiments. The outcomes, therefore, of the docking simulations and the inter-ligand NOEs provide a clear interpretation of the binding mode of the interaction of artemisinin with HSA. Moreover, it is clear that FA7 of HSA has a repertoire of amino acids and enough space to attract ligands with ionic, aromatic, apolar, or polar characteristics that bind in various spatial and interacting ways.

#### 2.3.2. Ibuprofen Binding Site (FA4)

**Figure 10:**
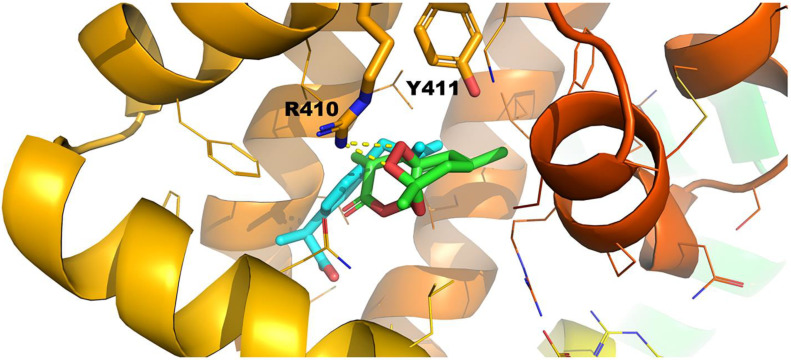
Superimposed pose number 1 of ibuprofen and artemisinin for binding site FA4 of HSA (1BXG.pdb).

The affinities of artemisinin and ibuprofen for FA4 were almost identical regarding the results when the 1BM0 structure was employed. On the contrary, when the 2BXG structure was used, a difference of 2.0 kcal/mol emerged in favor of ibuprofen. In Figure 10, pose number 1 for artemisinin and ibuprofen are superimposed. In general, the ionic interaction between ibuprofen and positively charged amino acids prevails in every case. The docking results are, therefore, in excellent agreement with the significant reduction in the NMR linewidths (Figure 5A,C and Figure S2A,C (Supplementary Material)) and STD integrals (Figure 6C,D) of the artemisinin resonances upon the addition of ibuprofen.

It would be of interest to compare selected poses of the docking calculations with experimental inter-ligand NOEs of Table S1. 14-CH_3_, 13-CH_3_, and 15-CH_3_ resonances show strong and strong-to-medium inter-NOEs, especially with the aromatic moiety of ibuprofen. This is in excellent agreement with the superposition of pose number 1 of ibuprofen and artemisinin (Figure 10), which shows that the respective functional groups have distances of <0.5 Å. 

## 3. Investigation of the Interaction of *Artemisia annua* Extract with BSA

**Figure 11:**
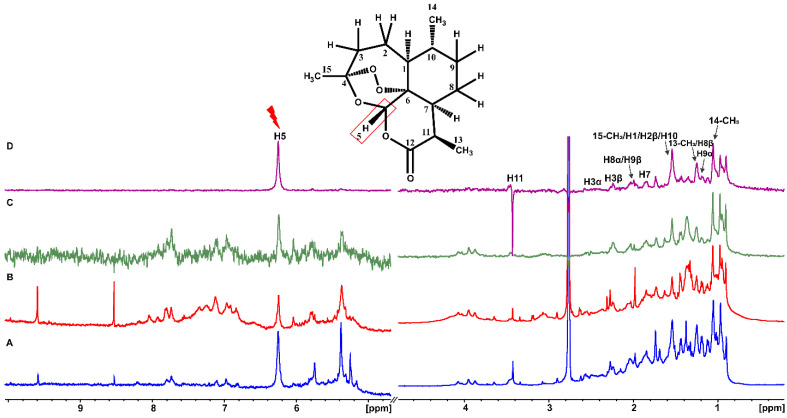
^1^H NMR of 2.6 mg *A. annua* without (**A**) and with 20 μM of BSA (**B**) in 50mM PBS buffer solution in D_2_O, pD 7.4 with 10% DMSO-d_6_. (**C**) STD NMR of sample B. (**D**) STD NMR of sample B with on-resonance irradiation at proton H-5 (6.26 ppm) of artemisinin. Τ = 310K, number of scans = 720, and total experimental time = 7 h 45 min.

*Artemisia annua* diethyl ether extract was previously analyzed by our group, and its major components were identified and quantified by NMR spectroscopy and mass spectrometry [37]. The ^1^H NMR spectrum of the above extract (Figure 11A) shows, upon the addition of 20 μM serum albumin (Figure 11B), considerable line broadening due to an increase in transverse relaxation rate of several analyte peaks, such as artemisinin, arteannuin, chrysosplenetin, and chrysosplenol D. This result indicates binding to BSA. Figure 11C shows that the most characteristic H5 resonance of artemisinin (δ = 6.26 ppm) shows the strongest STD signal. 

This confirms that artemisinin is the principal analyte of *A. annua* and is involved in the interaction with BSA. However, the region between 1 and 4 ppm is rather overcrowded; therefore, the clear discrimination of artemisinin STD resonances is a challenging issue. To overcome this problem, we applied a reverse STD experiment and performed the selective irradiation of the most characteristic H-5 resonance of artemisinin (Figure 11D). Interestingly, magnetization was successfully transferred only to the resonances of artemisinin, thereby, allowing the identification of discrete STD signals of artemisinin. 

## 4. Materials and Methods

### 4.1. Chemicals and Standards 

Artemisinin (98%), warfarin, and ibuprofen (98%), were purchased from Sigma Aldrich (Schnelldorf, Germany).

### 4.2. Artemisia Annua Extraction 

We used 100 mL of diethyl ether to extract 4 g of *A. annua* plant sample (obtained from Teemana, Germany) in a sonication bath and kept this cold with ice for 1 h. The solvent was evaporated using a rotary evaporator, and the dry extract was kept in a flask in refrigeration.

### 4.3. NMR Methodologies

STD and Tr-NOESY NMR experiments were performed at 37 °C on a Bruker AV-500 spectrometer (Bruker Biospin, Rheinstetten, Germany). BSA (20 μM) and 50 mM PBS (pD 7.4) in D_2_O with 10% DMSO-*d*_6_ was used to facilitate the dissolution of artemisinin (2 mM) and the drugs warfarin and ibuprofen. Artemisinin, warfarin, and ibuprofen were first dissolved in DMSO-*d_6_* and then diluted in PBS. 

The solutions were transferred into a 5 mm NMR tube. For the competition experiments, the selective drugs warfarin and ibuprofen were added to the solution in molar concentrations as indicated in the Figure captions. The on-resonance irradiation of the protein was performed at 4.2 ppm. Off-resonance irradiation was applied at −40 ppm. Each Gaussian pulse required 50 ms separated by a delay of 1 ms. The saturation time was set at 2 s. An excitation sculpting pulse sequence was used for water suppression. 

Tr-NOESY NMR spectra were acquired (56 scans and a 2k data block) with 110 incremental values of the evolution time (States-TPPI). The total experimental time was 4 h. Solvent suppression was achieved using an excitation sculpting scheme. INPHARMA NMR competition experiments were performed as in the case of Tr-NOESY experiments with mixing times of 100, 200, and 300 ms.

### 4.4. Computational Methods

Since the binding sites of warfarin (FA7) and ibuprofen (FA4) are known from single crystal X-ray structure determination [20,21,22,23,33,37] and artemisinin is shown from our experimental INPHARMA NMR data to be competitive towards FA7 and FA4, local docking was performed to identify the strength of interaction and the anchoring sites. The human serum albumin crystal structures were downloaded from PDB. The entry code names of the structures are: 1BM0 (HSA free of ligands) [36], 2BXD (HSA complexed with warfarin) [21], and 2BXG (HSA complexed with ibuprofen) [21]. 

Warfarin and ibuprofen, in their deprotonated forms, and neutral artemisinin were built with gaussview 6.0.16.3 [38]. The ligands were optimized at the DFT-CAM-B3LYP/aug-cc-pvdz level of theory and basis set. For the molecular docking of the above molecules to HSA, the AutoDock Vina1.1.24 [39] software package was used since it is one of the fastest and more accurate software tools for this method [40]. AutoDock Tools 1.5.6 [41] was used as a preprocessing software package to prepare the protein and docking simulation (the addition of polar hydrogens, deletion of existing cocrystallized ligands and water molecules, the definition of the dihedral angles of the ligand (which were allowed to vary), and the definition of the search space). 

For binding site FA7, site-specific docking was performed with increased exhaustiveness for the following protein-ligand pairs: 1BM0–artemisinin, 1BM0–warfarin, 2BXD–artemisinin, and 2BXD–warfarin. The binding site FA7 (subdomain IIA) is characterized by the amino acids K199, R218, R222, H242, and R257, while the binding site FA4 (subdomain IIIA) includes the amino acids R410, Y411, S489, S419, and T422. For binding site FA4, ibuprofen and artemisinin were docked to 1BM0 and 2BXD, resulting in the pairs 1BM0–artemisinin, 1BM0–ibuprofen, 2BXD–artemisinin, and 2BXD–ibuprofen. The procedure is identical to our previous work [33] following the self-docking protocol. Each docking simulation consisted of 10 independent runs for each complex. The docking conformations were selected based on the highest binding affinity and in accordance with our experimental data.

## 5. Conclusions

The combined strategy of saturation transfer difference (STD), transfer nuclear Overhauser effect spectroscopy (TR-NOESY), STD–total correlation spectroscopy (STD-TOCSY), and Inter-ligand Noes for PHArmacophore Mapping (INPHARMA) NMR methods and molecular docking calculations was shown to be useful to investigate the structural basis of the interaction of artemisinin with HSA/BSA. The significant number of inter-NOE connectivities in the INPHARMA competition experiments of artemisinin with the drugs warfarin and ibuprofen demonstrated, unequivocally, that they share common FA7 and FA4 binding sites (≤5 Å) and, thus, are competitive rather than allosteric. 

The INPHARMA technique in conjunction with docking calculations can elucidate the molecular basis of interactions between natural products and synthetic analogues [33,34,42] with serum albumin. Artemisinin was shown to be responsible for the main interaction of the *A. annua* extract with BSA. Further NMR and computational studies are currently in progress to investigate the interaction of artemisinin derivatives and hydroperoxide biosynthetic precursors [43,44], both in isolated form as well as in complex plant extracts, with their macromolecular targets.

## Data Availability

The data presented in this study are available in supplementary material.

## Supplementary Materials

The following supporting information can be downloaded at: https://www.mdpi.com/article/10.3390/molecules27185912/s1, Figure S1: (A) ^1^H NMR of 2 mM artemisinin in PBS, pD 7.4, D_2_O with 10% DMSO-d_6_. (B) ^1^H NMR of 2 mM artemisinin with 20 μM HSA in PBS, pD 7.4, D_2_O with 10% DMSO-d_6_. (C) STD NMR of 2 mM artemisinin with 20 μM HSA. Figure S2: (A) Selected regions of ^1^H NMR of 2 mM artemisinin with 20 μM HSA in PBS, pD 7.4, D_2_O with 10% DMSO-d_6_, (B) after the addition of 2 mM warfarin, and (C) after the addition of 1 mM ibuprofen. Figure S3: (A) ^1^H NMR of 2 mM artemisinin and 1.8 mM of warfarin with 20 μM of HSA in PBS buffer solution in D_2_O pD 7.4 with 10% DMSO-d_6_. (B) STD NMR of sample A. (C) ^1^H NMR of 2 mM artemisinin, 1 mM of ibuprofen and 20 μM of HSA in PBS buffer solution pD 7.4 in D_2_O with 10% DMSO-d_6_. (D) STD NMR of sample C. Τ = 310, number of scans = 320, and total experimental time = 3 h 30 min. Figure S4: Selective region of 2D Tr-NOESY NMR spectrum (d8 = 300 ms) of 2 mM artemisinin with 20 μM HSA after the addition of 2 mM warfarin in PBS buffer solution in D_2_O, pD 7.4 with 10% DMSO-d_6_. (A) Red cross-peaks correspond to inter-NOEs between warfarin and artemisinin and (B) blue cross-peaks correspond to intra-NOEs of artemisinin. Mixing time = 300 ms, T = 310 K, number of scans = 112, and experimental time = 17 h 19 min. Figure S5: Selective regions of 2D Tr-NOESY NMR spectrum (d8 = 300 ms) of 2 mM artemisinin and 20 μM HSA after the addition of 1.2 mM ibuprofen in PBS buffer solution in D_2_O, pD 7.4 with 10% DMSO-d_6_. Blue cross-peaks correspond to intra-NOEs, and red cross-peaks correspond to inter-NOEs. Mixing time = 300 ms, T = 310 K, number of scans = 112, and experimental time = 17 h 19 min. Table S1: Inter-NOEs between warfarin and artemisinin and ibuprofen and artemisinin in BSA/HSA.

## Sample Availability

Samples of the compounds and plant extracts are available from the authors.
