# Supplementary material for: Structural Basis of Artemisinin Binding Sites in Serum Albumin with the Combined Use of NMR and Docking Calculations
Source: Molecules. 2022 Sep 12;27(18):5912. doi: 10.3390/molecules27185912 (PMC9506303; doi:10.3390/molecules27185912)
Supplement: Supplementary file 1 [file molecules-27-05912-s001.zip › molecules-1882982-supplementary.pdf]

Structural basis of artemisinin binding sites in serum albumin with the combined use of NMR and docking calculations

Alexandra Primikyri *, Georgios Papamokos, Themistoklis Venianakis, Marianna Sakka, Vassiliki Kontogianni and Ioannis P. Gerothanassis*

Section of Organic Chemistry and Biochemistry, Department of Chemistry, University of Ioannina, Greece
* Correspondence: igeroth@uoi.gr (I.P.G.); a.primikyri@uoi.gr (A.P.)

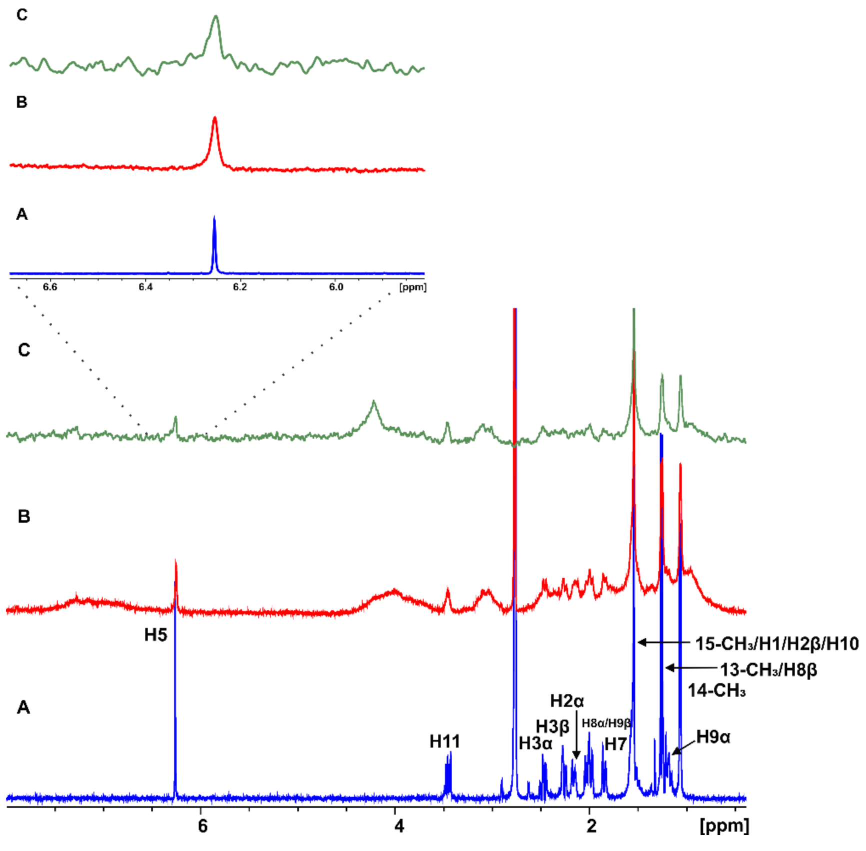

Figure S1. (A) ^{1}H NMR of 2mM artemisinin in PBS, pD 7.4, D_2O with 10% DMSO-d_6. (B) ^{1}H NMR of 2mM artemisinin with 20 µM HSA in PBS, pD 7.4, D_2O with 10% DMSO-d_6. (C) STD NMR of 2mM artemisinin with 20 µM HSA. T = 310K, number of scans = 80, total experimental time = 50min.

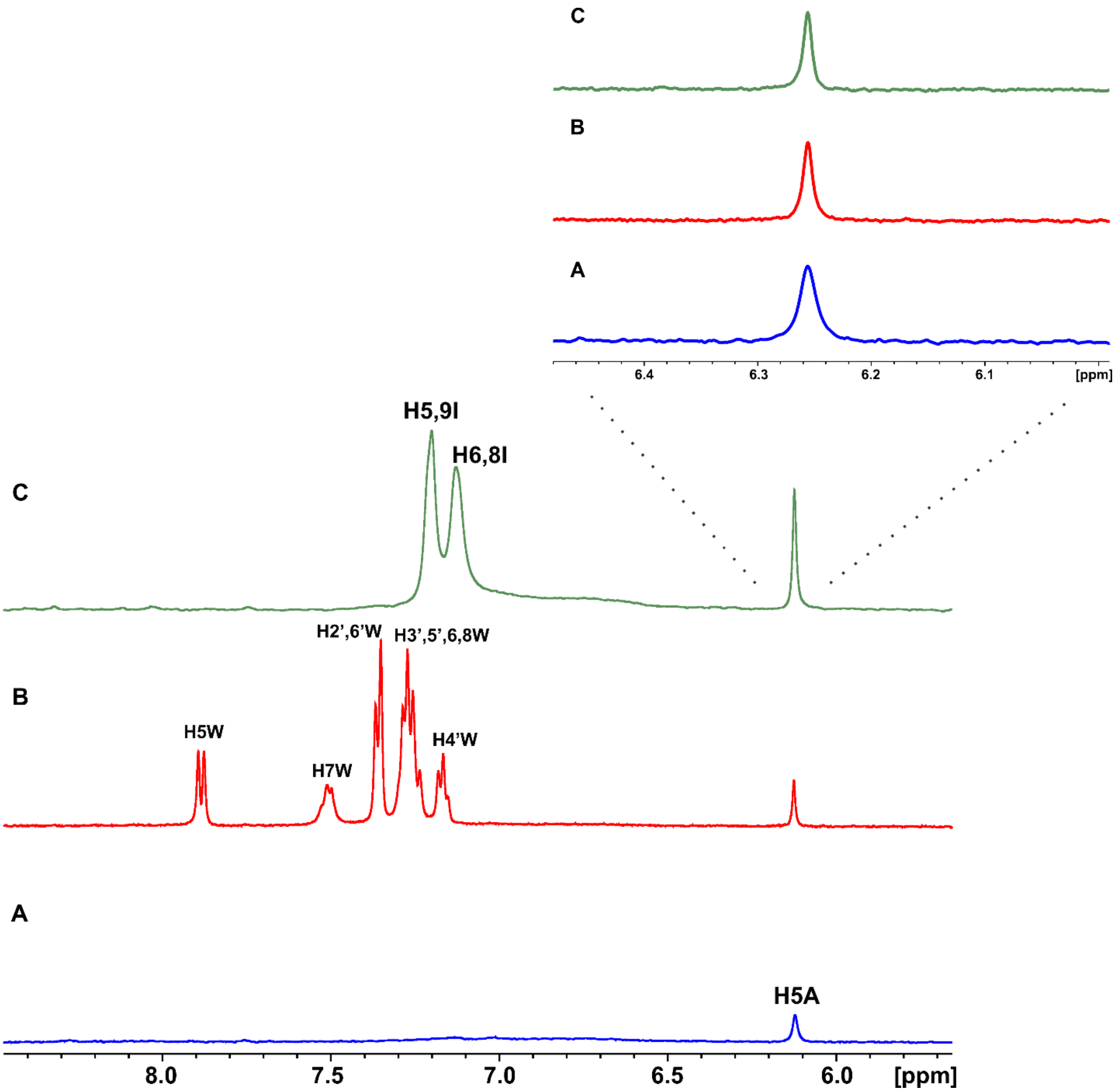

Figure S2. (A) Selected regions of ^{1}H NMR of 2mM artemisinin with 20 µM HSA in PBS, pD 7.4, D_2O with 10% DMSO-d_6, (B) after the addition of 2 mM warfarin and (C) After the addition of 1 mM ibuprofen.

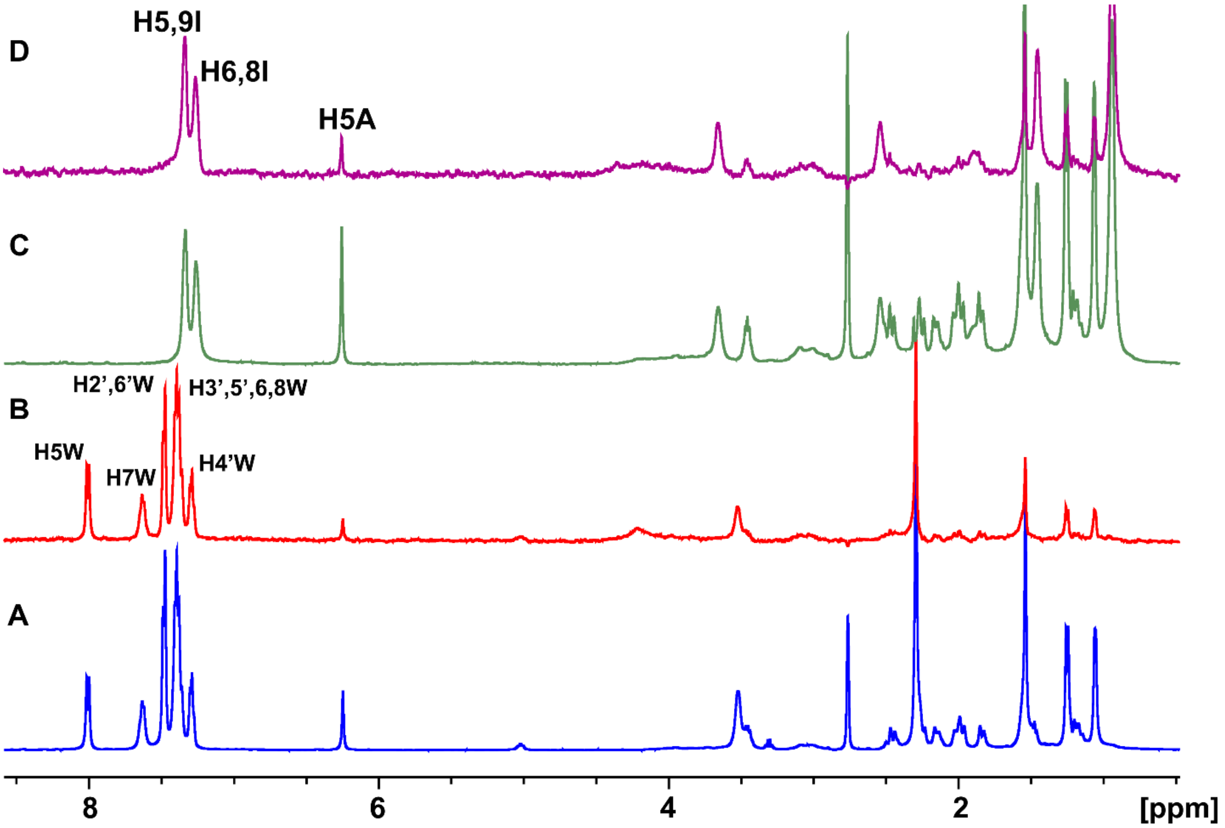

Figure S3. (A) ^{1}H NMR of 2 mM artemisinin and 1.8 mM of warfarin with 20 µM of HSA in PBS buffer solution in D_2O pD 7.4 with 10% DMSO-d_6. (B) STD NMR of sample A. (C) ^{1}H NMR of 2 mM artemisinin, 1 mM of ibuprofen and 20 µM of HSA in PBS buffer solution pD 7.4 in D_2O with 10% DMSO-d_6. (D) STD NMR of sample C. T = 310, number of scans =320, total experimental time = 3h30min.

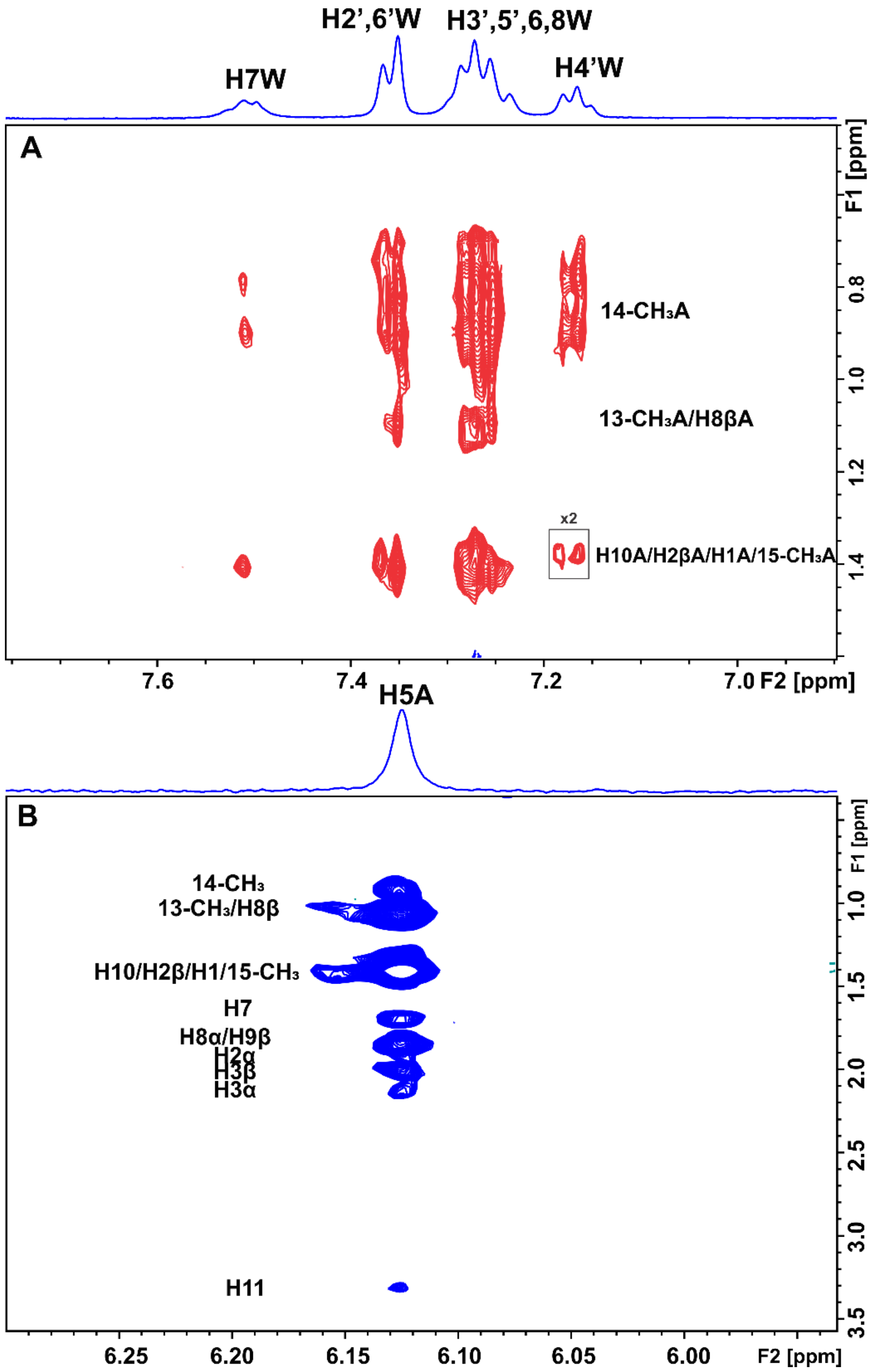

Figure S4. Selective region of 2D Tr-NOESY NMR spectrum (d8 = 300 ms) of 2 mM artemisinin and 20 µM HSA after the addition of 2 mM warfarin in PBS buffer solution in D_2O pD 7.4 with 10% DMSO-d_6. (A) Red cross-peaks correspond to inter-NOEs between warfarin and artemisinin and (B) blue cross-peaks correspond to intra-NOEs of artemisinin. Mixing time = 300 ms, T = 310 K, number of scans = 112, experimental time = 17h 19 min.

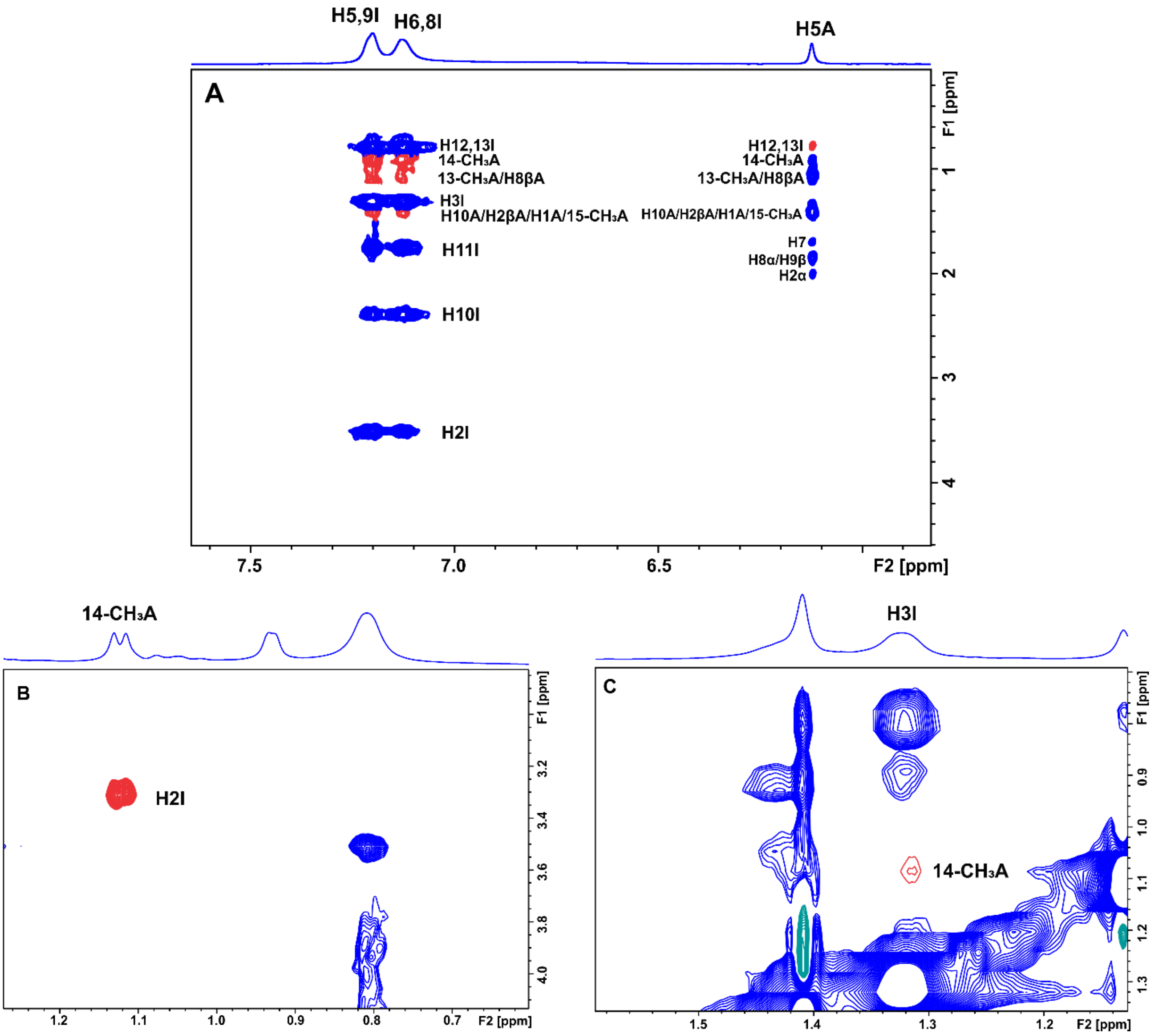

Figure S5. Selective regions of 2D Tr-NOESY NMR spectrum (d8 = 300 ms) of 2 mM artemisinin and 20 µM HSA after the addition of 1.2 mM ibuprofen in PBS buffer solution in D_2O pD 7.4 with 10% DMSO-d_6. Blue cross-peaks correspond to intra-NOEs and red cross-peaks to inter-NOEs. Mixing time = 300 ms, T = 310 K, number of scans = 112, experimental time = 17h 19 min.

Table S1. Inter-NOEs between warfarin and artemisinin and ibuprofen and artemisinin in complexation with BSA/HSA.

inter-NOEs in BSA				inter-NOEs in HSA		
**Warfarin**	**Artemisinin**	**Integral**	**Intensity**	**Artemisinin**	**Integral**	**Intensity**
H5	14-CH_3	0,148	strong	14-CH_3	0,058	strong-medium
				H10/H2β/H1/15-CH3	0,009	weak
H7	14-CH_3	0,115	strong-medium	14-CH_3	0,084	strong
				H10/H2β/H1/15-CH3	0,044	strong-medium
H2',6'	14-CH_3	0,176	strong	14-CH_3	0,152	strong
	13-CH_3/H8β	0,101	strong-medium	13-CH_3/H8β	0,039	strong-medium
	H10/H2β/H1/15-CH3	0,159	strong	H10/H2β/H1/15-CH3	0,080	strong
				14-CH_3	0,152	strong
H3',5',6,8	14-CH_3	0,250	strong	14-CH_3	0,250	strong
	13-CH_3/H8β	0,114	strong-medium	13-CH_3/H8β	0,082	strong
	H10/H2β/H1/15-CH3	0,130	strong-medium	H10/H2β/H1/15-CH3	0,104	strong
H4'	14-CH_3	0,225	strong	14-CH_3	0,290	strong
	13-CH_3/H8β	0,163	strong	H10/H2β/H1/15-CH3	0,075	strong
	H10/H2β/H1/15-CH3	0,066	weak			

inter-NOEs in BSA				inter-NOEs in HSA		
**Ibuprofen**	**Artemisinin**	**Integral**	**Intensity**	**Artemisinin**	**Integral**	**Intensity**
H5,9	14-CH_3	0,893	strong	14-CH_3	0,910	strong
	13-CH_3/H8β	0,258	strong	13-CH_3/H8β	0,230	strong
	H10/H2β/H1/15-CH3	1,000	strong	H10/H2β/H1/15-CH3	0,277	strong
H6,8	14-CH_3	0,766	strong	14-CH_3	1,000	strong
	13-CH_3/H8β	0,153	strong-medium	13-CH_3/H8β	0,139	strong-medium
				H10/H2β/H1/15-CH3	0,220	strong
H12,13	H5	0,141	strong-medium	H5	0,043	weak
H11	H5	0,224	strong			
H2	14-CH_3	0,063	weak	14-CH_3	0,459	strong
H3	14-CH_3	0,841	strong	14-CH_3	0,374	strong
